# Does ulinastatin really reduce incidence of acute kidney injury after cardiac surgery?

**DOI:** 10.1186/s13054-016-1279-4

**Published:** 2016-04-26

**Authors:** Fu-Shan Xue, Gao-Pu Liu, Chao Sun

**Affiliations:** Department of Anesthesiology, Plastic Surgery Hospital, Chinese Academy of Medical Sciences and Peking Union Medical College, Beijing, 100144 People’s Republic of China

In a retrospective analysis by Wan and colleagues [1], propensity score matching analysis showed that ulinastatin administration was associated with a decreased incidence of acute kidney injury (AKI) after cardiac surgery. Paradoxically, patient outcomes, including intensive care unit (ICU) length of stay, in-hospital length of stay, and mortality, were not significantly different between the ulinastatin and control groups, although AKI has been significantly associated with increased morbidity and mortality after cardiac surgery [2].

An issue with this study is that it did not report cardiovascular medicine administration. It has been shown that preoperative statin is associated with a reduced risk of postoperative AKI and mortality in patients undergoing elective cardiac surgery [3]. However, preopevrative use of renin-angiotensin system inhibitors has been associated with a 28 % increase in AKI after cardiac surgery and this effect is independent of intraoperative and postoperative hypotension, suggesting a role for the changes in glomerular capillary pressure induced by these drugs [4]. Similarly, it was unclear whether the two groups were comparable with respect to types of cardiac surgery. The risk of AKI and mortality after cardiac surgery increases progressively with complexity of the planned procedure, i.e., the risk is lowest in patients undergoing coronary artery bypass grafting only, while it increases after valve replacement surgery and is greatest after combined coronary artery bypass grafting and valve procedures [5]. We are concerned that the lack of inclusion of these risk factors in the propensity score matching model would have biased the effect of ulinastatin administration on postoperative AKI and mortality in this study.

## Abbreviations

AKI: acute kidney injury
CSA: cardiac surgery-associated
ICU: intensive care unit
