# Supplementary material for: Does ulinastatin really reduce incidence of acute kidney injury after cardiac surgery?
Source: Crit Care. 2016 Apr 26;20:112. doi: 10.1186/s13054-016-1279-4 (PMC4845367; doi:10.1186/s13054-016-1279-4)
Supplement: Related texts

# Reply: Authors’ response

**Authors:** Xin Wan, Xiangcheng Xie, Yasser Gendoo, Xin Chen, Xiaobing Ji, Changchun Cao

We appreciate the valuable comments by Dr. Xue and colleagues regarding our article and agree that perioperative medications may in fact be involved in the development of AKI.

The protective role of statins remains a controversial issue. On one hand, they have been shown to possess anti-inflammatory properties by effectively reducing inflammatory cytokines. However, a systemic review containing seven randomized controlled trials did not find evidence that preoperative statin use is associated with a reduction in the incidence of AKI after cardiac surgery [6]. This issue has also been discussed in *Critical Care* [7].

In our study, we initially took medications into account, which included statins, phosphocreatine, nonsteroidal anti-inflammatory drugs, hydroxyethyl starch, and mannitol. Using multiple logistic regression analysis, our results showed no obvious protective effect of the phosphocreatine and statins in reducing the risk of AKI (data not shown). However, we found that ulinastatin played a protective role in the development of cardiac surgery-associated (CSA)-AKI after adjusting for statins and other factors. Therefore, we further performed propensity matching to confirm the result.

To date, the risk factors for AKI have been well studied [8]. We do agree that the risk of developing AKI is associated with the types of cardiac surgery and the complexity of the operation, which includes variables such as different cardiopulmonary bypass duration, hypothermia, mean artery pressure, transfusion, hematocrit, and so forth. These cardinal possible risk factors were considered and included in our study design, thus making the two groups relatively comparable. In addition, because this is a retrospective study, other medications which may also have had an impact on the development of AKI, such as antibiotics, diuretics, steroids, angiotensin converting enzyme inhibitors/angiotensin-receptor blockers, and so forth, were not analyzed. We do acknowledge the possible existence of bias, and thus prospective, randomized, controlled trials with a large sample size and perioperative administration of ulinastatin are needed to confirm the reno-protective effect of ulinastatin in the development of CSA-AKI.
